# Circ-DONSON Knockdown Inhibits Cell Proliferation and Radioresistance of Breast Cancer Cells via Regulating SOX4

**DOI:** 10.1155/2021/8461740

**Published:** 2021-11-22

**Authors:** Xiufang Zhu, Lei Li

**Affiliations:** Department of Medical Imaging, The First People's Hospital of Lianyungang, Lianyungang, Jiangsu 222002, China

## Abstract

**Background:**

Circular RNAs have been validated as critical regulators in the development of breast cancer (BC). Circ-DONSON is involved in the progression of glioma and gastric cancer. However, the biological role of circ-DONSON in BC remains unclear, and the aim of this study was to explore the biological role of circ-DONSON in BC.

**Methods:**

Human tissue samples and BC cell lines were collected in this study. siRNAs against circ-DONSON were transfected into BC cell lines for silencing of circ-DONSON. Quantitative real-time PCR was used to test the circ-DONSON expression. Cell counting kit-8 (CCK-8), 5-bromo-2′ deoxyuridine enzyme-linked immunosorbent assay (BrdU-ELISA), colony formation, and caspase-3 activity assays were used to assess cell proliferation, cell survival, and cell viability. Western blotting analysis was used to detect the protein expression levels.

**Results:**

Our findings showed that circ-DONSON showed high expression in BC tissues and cell lines. CCK-8 and BrdU-ELISA assays showed that circ-DONSON knockdown inhibited BC cell proliferation. Moreover, cell survival, cell viability, and caspase-3 activity assays showed that circ-DONSON knockdown reduced the radioresistance of BC cells. Mechanistically, circ-DONSON regulated BC cell proliferation and radioresistance via SRY-box transcription factor 4 (SOX4). SOX4 overexpression significantly rescued the effect of circ-DONSON knockdown on BC cell proliferation and radioresistance. Moreover, circ-DONSON activated the Wnt/*β*-catenin pathway in BC cells via SOX4.

**Conclusion:**

Our study concluded that circ-DONSON knockdown hindered cell proliferation and radioresistance through the SOX4/Wnt/*β*-catenin pathway in BC.

## 1. Introduction

Breast cancer (BC) is one of the most common cancers in women worldwide [1, 2]. Although radiotherapy is a main treatment for BC patients, radioresistance leads to limited therapeutic efficacy [3, 4]. Therefore, it is urgently required to explore the molecular mechanisms of BC progression and radioresistance.

As a recently discovered member of the noncoding RNA family, circular RNAs (circRNAs) are characterized by a covalently closed continuous loop structure [5]. It has been reported that circRNAs are highly expressed in multiple tumors, such as colorectal cancer, lung cancer, gastric cancer, and BC [6, 7]. For example, circRNA_102171 silencing suppresses cell proliferation, migration, and invasion while promoting apoptosis of papillary thyroid cancer cells through modulating CTNNBIP1-dependent activation of the *β*-catenin pathway [8]. Hsa_circRNA_103809 regulates the colorectal cancer cell proliferation and migration via the miR-532-3p/FOXO4 axis [9]. In BC, circTADA2As suppresses cell proliferation, migration, and invasion via targeting the miR-203a-3p/SOCS3 axis [10]. However, more circRNAs involved in BC progression and therapy are needed to be explored.

Circ-DONSON has been reported to be highly expressed in glioma tissues and promote cell proliferation and migration through modulating FOXO3 [11]. Circ-DONSON is upregulated in gastric cancer tissues and positively correlated with poor prognosis of this cancer [12]. Circ-DONSON promotes gastric cancer cell growth and invasion via NURF complex dependent activation of SRY-box transcription factor 4 (SOX4) [12]. Moreover, linear DONSON is predicted by bioinformatics analysis to be upregulated in breast cancer. However, the biological function of circ-DONSON in BC remains unclear.

Circ-DONSON and linear DONSON are derived from the same pre-mRNA. We hypothesized that circ-DONSON is also upregulated in BC and serves as an oncogene. In this study, we found that circ-DONSON showed high expression in BC tissues and cell lines. Circ-DONSON knockdown inhibited cell proliferation and reduced the radioresistance of BC cells. Mechanistically, circ-DONSON-mediated biological functions in BC were dependent on SOX4. Moreover, circ-DONSON modulated the Wnt/*β*-catenin pathway via SOX4 in BC. In summary, circ-DONSON contributed to cell proliferation and radioresistance through the SOX4/Wnt/*β*-catenin pathway in BC and might be a potential therapeutic target for this disease.

## 2. Materials and Methods

### 2.1. Tissue Samples

Thirty paired human BC tissues and corresponding noncancerous tissues which are 5 cm away from tumor tissues were collected from patients who underwent surgical resection at the First People's Hospital of Lianyungang. The experiments were approved by the Ethics Committee of the First People's Hospital of Lianyungang. Written consent for this research purpose was obtained from each patient.

### 2.2. Cell Culture

A noncancerous breast epithelial cell (MCF-10A) and BC cell lines (MCF-7, MDA-MB-231, BT474, SKBR3, and BT549) were purchased from the Cell Bank of Chinese Academy of Sciences (Shanghai, China). The cells were maintained in DMEM (Gibco, Waltham, MA, USA) or RPMI-1640 (Gibco) medium with 10% (v/v) fetal bovine serum (Hyclone, Logan, UT), 100 U/ml penicillin and 100 U/ml streptomycin at 37°C with an atmosphere of 5% CO_2_ in a humidified cell chamber.

### 2.3. Quantitative Real-Time PCR (qRT-PCR)

Total RNA was extracted from tissues and cell lines using TRIzol reagent (Thermo Fisher Scientific, Waltham, MA, USA) according to the provider's protocol. The total RNA was reverse transcribed into cDNA using a PrimeScript™ RT Reagent Kit (Takara, Dalian, China). The qRT-PCR was performed using SYBR Green Master Mix (Vazyme, Nanjing, China) as described previously [13]. Primer sequences used in this study were as follows [11]: circ-DONSON, F: 5′-CCACATCGCGCTGGTTACGTC-3′, R: 5′-GACTACGATCGTCGTCAAGGCA-3'; GAPDH: F: 5′- TCATTTCCTGGTATGACAACGA-3′, R: 5′- GTCTTACTCCTTGGAGGCC-3'.

### 2.4. Cell Transfection

Circ-DONSON siRNAs and negative control (NC) si-NC were obtained from GenePharma (Shanghai, China). The pcDNA3.1-SOX4 and control empty vector were obtained from Genomeditech (Shanghai, China). Both siRNAs and vectors were transfected into the MCF-7 or MDA-MB-231 cells using Lipofectamine 3000 (Invitrogen) according to the manufacturer's instructions.

### 2.5. Cell Survival Assay

Cell survival was determined with a colony formation assay as described previously [14]. In briefly, equal numbers of cells were seeded into 6-well plates and then exposed to 0, 2, 4, 6, or 8 Gy X-ray irradiation. After 15 days, the cell colonies were stained with crystal violet. The number of colonies with cells >50 was counted. The survival fraction was calculated as the surviving colony fraction (colonies counted/total cells seeded) of the treatment plates divided by that of the control group. The cell survival curves were then fitted using a single hit multitarget model.

### 2.6. Cell Counting Kit-8 (CCK-8) Assay

CCK-8 (Dojindo, Kumamoto, Japan) was used to examine cell proliferation. Cells (5 × 10^3^) were plated into 96-well plates and stained with 10 *μ*L of sterile CCK8 solution at 2, 24, 48, and 72 h. The optical density (OD) was measured at a wavelength of 450 nm.

CCK-8 was also used to test the sensitivity of cells to radiotherapy. Cells were plated into 96-well plates and exposed to 4 Gy X-ray irradiation. After 24 h, CCK8 solution was added to each well, and the optical density (OD) was measured at a wavelength of 450 nm.

### 2.7. 5-Bromo-2′-deoxyuridine Enzyme-Linked Immunosorbent Assay (BrdU-ELISA)

We also used a Cell Proliferation ELISA-BrdU (colorimetric) kit (Roche Diagnostics, USA) to detect cell proliferation according to the manufacturer's instructions.

### 2.8. Caspase-3 Activity Assay

The caspase-3 activity of cells was assayed using the kit (Beyotime, Shanghai, China) according to the manufacturer's instructions.

### 2.9. Western Blot

We performed western blot analysis to detect the protein expression level as previously described [15]. The antibodies used in this study were as follows: anti-SOX4 (PA5-72852; 2 *µ*g/mL; Invitrogen), anti-Wnt1 (ab15251; 1 : 1000; Abcam), anti-*β*-catenin (ab68183; 1 : 500; Abcam), anticyclin B1 (ab32053; 1 : 50000; Abcam), anticyclin E1 (ab33911; 1 : 1000; Abcam), anti-CDK2 (ab32147; 1 : 1000; Abcam), and anti-GAPDH (ab8245; 1 : 500; Abcam).

### 2.10. Cell Cycle Analysis

A cell cycle detection kit (Keygen, Nanjing, Jiangsu, China) was used for cell cycle analysis according to the manufacturer's instructions. Cells were then analyzed using the ACEA NovoCyte system (ACEA Biosciences, Santa Clara, CA, USA).

### 2.11. Statistical Analysis

The data are presented as the mean ± standard deviation (SD). Student's *t*-test was used for statistical analysis. *P* value < 0.05 was considered statistically significant.

## 3. Results

### 3.1. Circ-DONSON Was Highly Expressed in BC Tissues and Cell Lines

**Figure 1:**
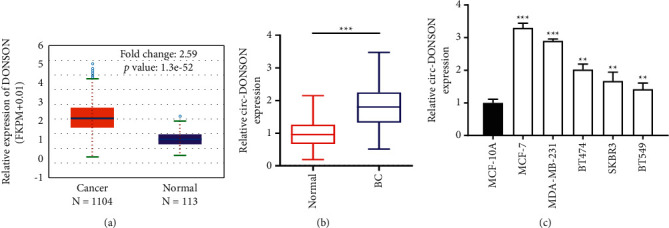
Circ-DONSON expression was increased in BC tissues and cell lines. (a) Expression of DONSON in 1104 BC tissues and 113 normal tissues was obtained from the ENCORI database. (b) Expression of circ-DONSON in BC tissues (*n* = 30) and adjacent normal tissues (*n* = 30). (c) Expression of circ-DONSON in noncancerous breast epithelial cells (MCF-10A) and BC cell lines (MCF-7, MDA-MB-231, BT474, SKBR3, and BT549). ^*∗∗*^*P* < 0.01 and ^*∗∗∗*^*P* < 0.001.

According to ENCORI database (https://starbase.sysu.edu.cn/), DONSON shows significant high expression in BC tissues compared to that in control tissues (Figure 1(a)). As shown in Figure 1(b), the levels of circ-DONSON in BC tissues were higher than that in corresponding noncancerous tissues. Moreover, compared with noncancerous breast epithelial cell MCF-10A, the levels of circ-DONSON in BC cell lines (MCF-7, MDA-MB-231, BT474, SKBR3, and BT549) were significantly upregulated (Figure 1(c)). Since MCF-7 and MDA-MB-231 cell lines contained relatively high expression of circ-DONSON, they were used for the subsequent assays.

Circ-DONSON knockdown inhibited BC cell proliferation, reduced the radioresistance of BC cells, and inhibited SOX4 expression.

**Figure 2:**
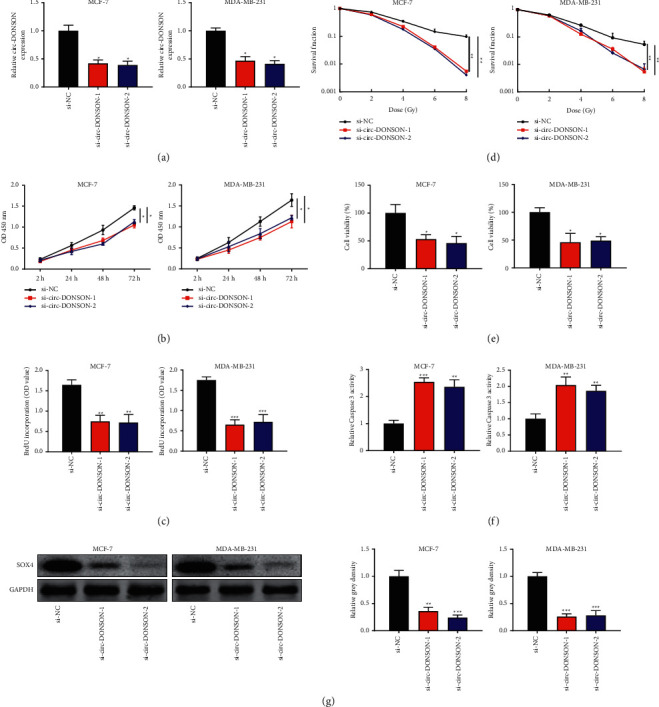
Circ-DONSON knockdown inhibited BC cell proliferation, reduced the radioresistance of BC cells, and inhibited SOX4 expression. (a) The expression of circ-DONSON in MCF-7 and MDA-MB-231 cells after treatment with circ-DONSON siRNAs. (b) CCK-8 assay was performed on MCF-7 and MDA-MB-231 cells after transfection with circ-DONSON siRNAs. (c) BrdU-ELISA assay was conducted on MCF-7 and MDA-MB-231 cells after transfection with circ-DONSON siRNAs. (d) Cell survival assay was performed on MCF-7 and MDA-MB-231 cells after exposure to 0, 2, 4, 6, or 8 Gy X-ray irradiation and transfection of circ-DONSON siRNAs. (e) Cell viability of MCF-7 and MDA-MB-231 cells after exposure to 4 Gy X-ray irradiation and transfection of circ-DONSON siRNAs was analyzed by CCK-8 assay. (f) The relative caspase-3 viability of MCF-7 and MDA-MB-231 cells after exposure to 4 Gy X-ray irradiation and treatment of circ-DONSON siRNAs. (g) The protein levels of SOX4 in MCF-7 and MDA-MB-231 cells after treatment with circ-DONSON siRNAs. The density of western blots bands was analyzed by ImageJ software. ^*∗*^*P* < 0.05, ^*∗∗*^*P* < 0.01, and ^*∗∗∗*^*P* < 0.001.

We knocked down circ-DONSON in MCF-7 and MDA-MB-231 cells with two circ-DONSON siRNAs (Figure 2(a)). The results of the CCK-8 assay showed that circ-DONSON siRNA treatment significantly reduced the proliferation ability of MCF and MDA-MB-231 cells (Figure 2(b)). Moreover, the BrdU-ELISA assay demonstrated that circ-DONSON knockdown inhibited the proliferation of MCF-7 and MDA-MB-231 cells (Figure 2(c)). As shown in Figure 2(d) (pictures are provided in Supplementary Figure 1), compared with si-NC, circ-DONSON siRNAs reduced the clonogenic survival of MCF-7 and MDA-MB-231 cells after irradiation. Irradiation dose at 4 Gy had a much more significant effect on cell proliferation than 2 Gy and did not cause too much damage to cells. Thus, radiation at the dose of 4 Gy was used for following experiments. Circ-DONSON siRNA treatment inhibited the cell viability of MCF-7 and MDA-MB-231 cells after irradiation (Figure 2(e)). Moreover, the caspase-3 activity assay showed that circ-DONSON knockdown increased apoptosis of MCF-7 and MDA-MB-231 cells after radiation exposure (Figure 2(f)). A previous report showed that circ-DONSON regulates SOX4 expression and facilitates gastric cancer cell growth and invasion [12]. Hence, we hypothesized that circ-DONSON promotes cell proliferation and radioresistance through the regulation of SOX4 in BC. As shown in Figure 2(g), results of western blot analysis showed that circ-DONSON siRNA treatment significantly reduced the protein expression of SOX4 in MCF-7 and MDA-MB-231 cells.

Circ-DONSON knockdown inhibited BC cell proliferation and reduced the radioresistance of BC cells via SOX4.

**Figure 3:**
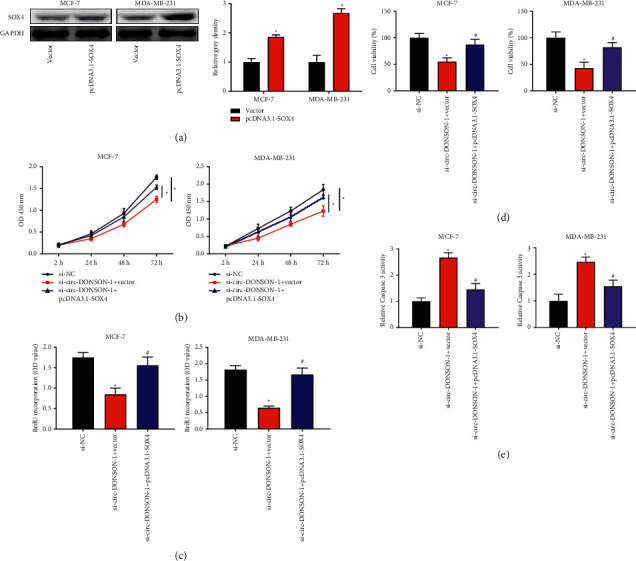
Circ-DONSON knockdown inhibited BC cell proliferation and reduced the radioresistance of BC cells via SOX4. (a) The protein levels of SOX4 in MCF-7 and MDA-MB-231 cells after treatment with pcDNA3.1-SOX4. The density of western blots bands was analyzed by ImageJ software. (b) CCK-8 assay was conducted on MCF-7 and MDA-MB-231 cells after cotreatment with circ-DONSON siRNA-1 and pcDNA3.1-SOX4. (c) BrdU-ELISA assay was performed on MCF-7 and MDA-MB-231 cells after cotreatment with circ-DONSON siRNA-1 and pcDNA3.1-SOX4. (d) Cell viability of MCF-7 and MDA-MB-231 cells with cotransfection of circ-DONSON siRNA-1 and pcDNA3.1-SOX4 after exposure to 4 Gy X-ray irradiation was analyzed by CCK-8 assay. (e) Relative caspase-3 viability of MCF-7 and MDA-MB-231 cells with cotreatment of circ-DONSON siRNA-1 and pcDNA3.1-SOX4 after exposure to 4 Gy X-ray irradiation. ^*∗*^*P* < 0.05 vs. si-NC and ^#^*P* < 0.05 vs. si-circ-DONSON-1+vector.

We used the pcDNA3.1-SOX4 vector to explore the rescue effect of SOX4 on circ-DONSON in BC cells. As shown in Figure 3(a), the pcDNA3.1-SOX4 vector significantly upregulated the protein levels of SOX4 in MCF-7 and MDA-MB-231 cells. SOX4 overexpression significantly rescued the effect of circ-DONSON knockdown on BC cell proliferation (Figures 3(b) and 3(c)). As shown in Figure 3(d), SOX4 overexpression partially abolished the effect of circ-DONSON knockdown on the cell viability of MCF-7 and MDA-MB-231 cells after irradiation. Moreover, SOX4 overexpression partially abolished the effect of circ-DONSON knockdown on cell apoptosis of MCF-7 and MDA-MB-231 cells after irradiation (Figure 3(e)). Moreover, silencing of circ-DONSON increased the percentage of cells in the G1 phase and decreased that in the S phase. Such effect was rescued by SOX4 (Supplementary Figure 2(a)). Supplementary Figure 2(b) revealed that circ-DONSON knockdown reduced cell cycle proteins including cyclin B1, cyclin E1, and CDK2. SOX4 rescued the suppressive effects of circ-DONSON knockdown on these proteins.

Circ-DONSON activated the Wnt/*β*-catenin pathway in BC cells via SOX4.

**Figure 4:**
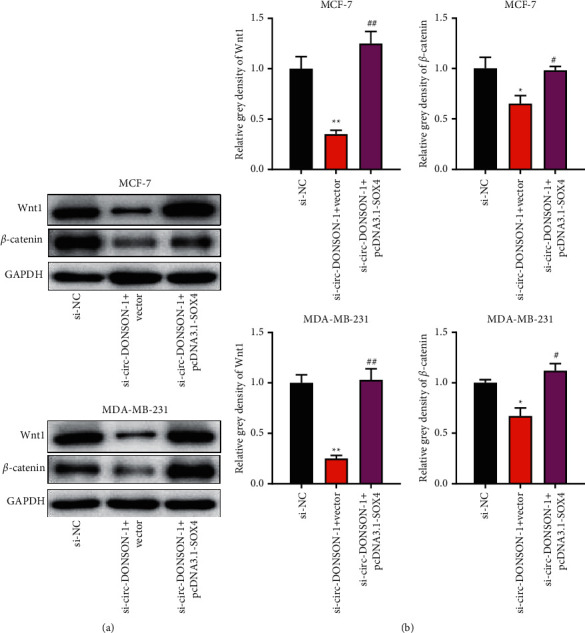
Circ-DONSON activated the Wnt/*β*-catenin pathway in BC cells via SOX4. (a) The protein levels of Wnt1 and *β*-catenin in MCF-7 and MDA-MB-231 cells that were cotreated with circ-DONSON siRNA-1 and pcDNA3.1-SOX4. The density of western blots bands shown in (b) is analyzed by ImageJ software. ^*∗*^*P* < 0.05 and ^*∗∗*^*P* < 0.01 vs. si-NC; ^#^*P* < 0.05 and ^##^*P* < 0.01 vs. si-circ-DONSON-1 + vector.

The Wnt/*β*-catenin pathway has been reported to be involved in cell proliferation and radioresistance of BC cells [16, 17]. Therefore, we investigated whether circ-DONSON activates the Wnt/*β*-catenin pathway in BC cells via SOX4. As shown in Figures 4(a) and 4(b), circ-DONSON knockdown significantly reduced the protein expression of Wnt1 and *β*-catenin in MCF-7 and MDA-MB-231 cells. Moreover, SOX4 overexpression rescued the effect of circ-DONSON knockdown on *β*-catenin in MCF-7 and MDA-MB-231 cells (Figures 4(a) and 4(b)).

## 4. Discussion

Several circRNAs have been reported to regulate BC progression. Tang et al. showed that hsa_circ_0001982 knockdown suppresses BC cell proliferation and invasion and induces apoptosis by targeting miR-143 [18]. Pan et al. found that knockdown of circ-TFF1 hinders BC cell proliferation, migration, invasion, and EMT *in vitro* and controls tumor growth *in vivo* through targeting the miR-326/TFF1 signaling [19]. Zhang et al. showed that hsa_circ_0072995 regulates the invasion and migration of BC cells through binding with miR-30c-2-3p [20]. Gao et al. reported that circ_0006528 could promote DNA synthesis and cell proliferation, invasion, and migration of BC cells by sponging miR-7-5p and activating the MAPK/ERK signaling pathway [21]. In this study, we found that circ-DONSON was highly expressed in BC tissues and cell lines. Moreover, circ-DONSON knockdown inhibited BC cell proliferation. These results suggest that circ-DONSON plays an oncogenic role in BC progression.

Previous studies also showed that circRNAs are involved in the radioresistance of cancers [22]. Circular RNA PRKCI knockdown inhibits esophageal cancer progression and elevates cell radiosensitivity through regulating the miR-186-5p/PARP9 axis [23]. Guan et al. showed that circPITX1 silencing represses glycolysis to enhance the radiosensitivity of glioma cells through the miR-329-3p/NEK2 axis [24]. The knockdown of circRNA_000543 sensitizes nasopharyngeal carcinoma to irradiation by targeting the miR-9/platelet-derived growth factor receptor B axis [25]. Liu et al. reported that circRNA_100367 attenuates radioresistance of esophageal squamous cell carcinomas cells through the miR-217/Wnt3 pathway [26]. In this study, we found that circ-DONSON knockdown reduced the clonogenic survival of BC cells and inhibited the cell viability of BC cells after irradiation. Moreover, circ-DONSON knockdown increased apoptosis of BC cells after radiation exposure. These results suggest that circ-DONSON knockdown reduces the radioresistance of BC cells.

As a member of the sex-determining region Y-related HMG box (SOX) transcription factor family, SOX4 is abnormally expressed in multiple cancers and exerts an oncogenic function [27]. The mRNA and protein levels of SOX4 are highly expressed in BC tissues compared with adjacent normal mammary tissues and positively correlated with the clinical stage [28]. Overexpression of SOX4 promotes epithelial-mesenchymal transition and stem cell characteristics of gastric cancer cells [29]. Koumangoye et al. showed that SOX4 promotes cell proliferation and invasion by silencing miR-31 via activation and stabilization of a corepressor complex with EZH2 and HDAC3 in esophageal cancer [30]. Ding et al. showed that circ-DONSON could regulate SOX4 expression and facilitate gastric cancer growth and invasion [12]. Therefore, we hypothesized that circ-DONSON promoted cell proliferation and radioresistance through regulating SOX4 in BC. Circ-DONSON knockdown significantly reduced the protein expression of SOX4 in MCF-7 and MDA-MB-231 cells. Moreover, SOX4 overexpression significantly rescued the effect of circ-DONSON knockdown on BC cell proliferation and radioresistance. These results suggest that circ-DONSON regulates cell proliferation and radioresistance of BC cells through SOX4.

The Wnt/*β*-catenin pathway has been reported to regulate tumorigenesis and progression of BC [31–33]. Li et al. showed that TRIM63, a novel oncogene, promotes BC cell proliferation and migration via activating the Wnt/*β*-catenin signaling pathway [34]. Also, the silencing of SOHLH2 enhances BC cell proliferation by activating the Wnt/*β*-catenin signaling pathway [35]. Given that SOX4 could stimulate *β*-catenin activity [36–38], we surmised that circ-DONSON could activate the Wnt/*β*-catenin signaling via SOX4. Circ-DONSON knockdown reduced the protein expression of Wnt1 and *β*-catenin in BC cells. Moreover, SOX4 overexpression abolished the effect of circ-DONSON knockdown on Wnt1 and *β*-catenin in BC cells. These results suggest that circ-DONSON activates the Wnt/*β*-catenin pathway in BC cells via SOX4.

To sum up, our findings demonstrated that circ-DONSON knockdown inhibits proliferation and reduces the radioresistance of BC cells through SOX4 and Wnt/*β*-catenin signaling.

## Data Availability

All data collection and analysis were conducted under double-blind and supported by the First People's Hospital of Lianyungang. The original data will be provided at any time if necessary.

## Supplementary Materials

Supplementary Materials
*Supplementary Figure 1*. Representative pictures of colony formation assay. *Supplementary Figure 2*. Effects of circ-DONSON/SOX4 on cell cycle. (a) Cell cycle analysis was performed in cells after transfection with si-NC or cotransfection with si-circ-DONSON-1 + empty pcDNA3.1 and si-circ-DONSON-1 + pcDNA3.1-SOX4. (b) Protein levels of cyclin B1, cyclin E1, and CDK2 in MCF-7 and MDA-MB-231 cells after indicated transfections were revealed by western blotting analysis. ^*∗*^*P* < 0.05 and ^*∗∗*^*P* < 0.01 vs. si-NC; ^#^*P* < 0.05 and ^##^*P* < 0.01 vs. si-circ-DONSON-1 + vector.
