# Supplementary material for: Circ-DONSON Knockdown Inhibits Cell Proliferation and Radioresistance of Breast Cancer Cells via Regulating SOX4
Source: J Oncol. 2021 Nov 22;2021:8461740. doi: 10.1155/2021/8461740 (PMC8629618; doi:10.1155/2021/8461740)
Supplement: Supplementary Materials — Supplementary Figure 1. Representative pictures of colony formation assay. Supplementary Figure 2. Effects of circ-DONSON/SOX4 on cell cycle. (a) Cell cycle analysis was performed in cells after transfection with si-NC or cotransfection with si-circ-DONSON-1 + empty pcDNA3.1 and si-circ-DONSON-1 + pcDNA3.1-SOX4. (b) Protein levels of cyclin B1, cyclin E1, and CDK2 in MCF-7 and MDA-MB-231 cells after indicated transfections were revealed by western blotting analysis. ∗P < 0.05 and ∗∗P < 0.01 vs. si-NC; #P < 0.05 and ##P < 0.01 vs. si-circ-DONSON-1 + vector. [file 8461740.f1.zip › 8461740.f1/Supplementary Figure 2.docx]

**
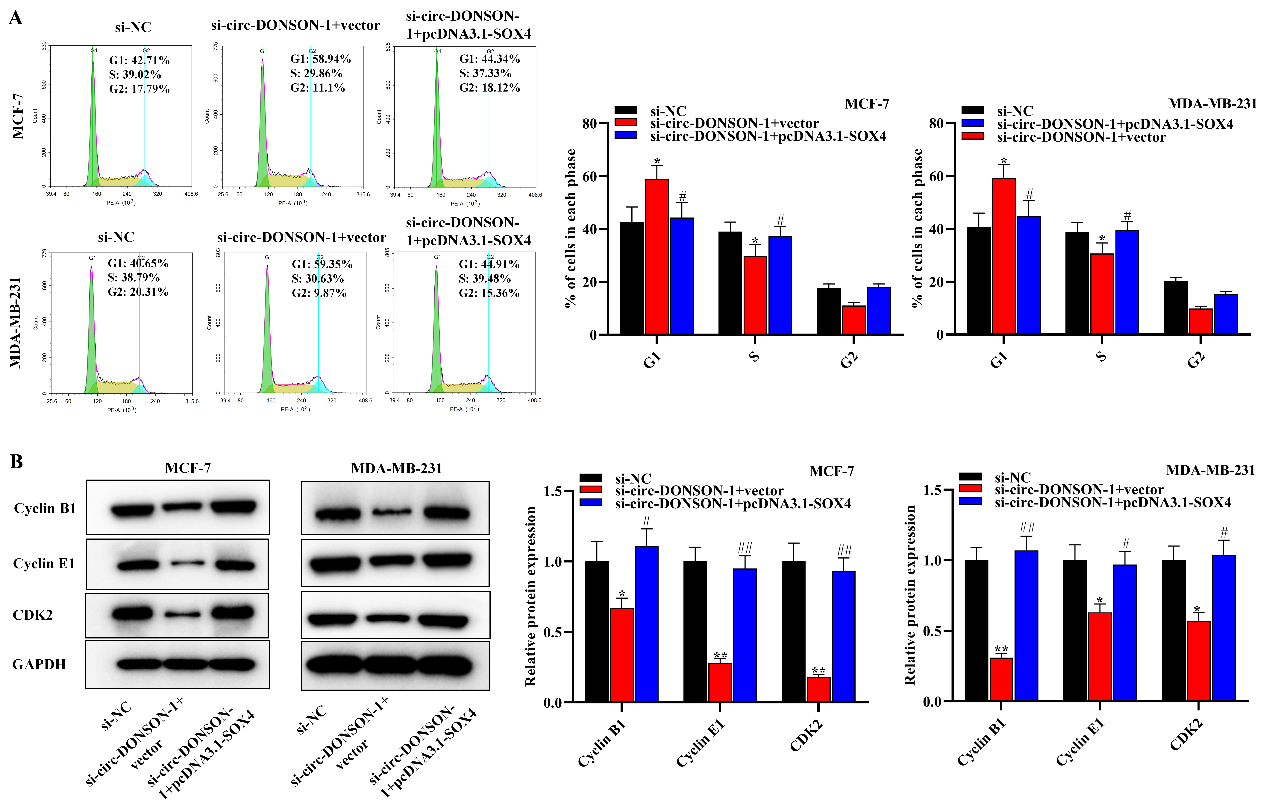
**

**Supplementary Figure 2. Effects of circ-DONSON/SOX4 on cell cycle.**

(A) Cell cycle analysis was performed in cells after transfection with si-NC, or cotransfection with si-circ-DONSON-1 + empty pcDNA3.1, si-circ-DONSON-1 + pcDNA3.1-SOX4. (B) Protein levels of Cyclin B1, Cyclin E1, CDK2 in MCF-7 and MDA-MB-231 cells after indicated transfections were revealed by western blotting analysis. **P* < 0.05, ***P* < 0.01 vs si-NC, ^#^*P* < 0.05, ^##^*P* < 0.01 vs si-circ-DONSON-1+vector.
